# Toward Versatile Transient Electronics: Electrospun Biocompatible Silk Fibroin/Carbon Quantum Dot-Based Green-Emission, Water-Soluble Piezoelectric Nanofibers

**DOI:** 10.3390/polym17111579

**Published:** 2025-06-05

**Authors:** Zhipei Xia, Chubao Liu, Juan Li, Biyao Huang, Chu Pan, Yu Lai, Zhu Liu, Dongling Wu, Sen Liang, Xuanlun Wang, Weiqing Yang, Jun Lu

**Affiliations:** 1 School of Chemistry, Southwest Jiaotong University, Chengdu 610031, China; 13981794626@163.com (Z.X.); 18202840217@163.com (J.L.); 13164627201@163.com (B.H.); panchu@my.swjtu.edu.cn (C.P.); wdl4186@my.swjtu.edu.cn (D.W.); 15034167632@163.com (S.L.); 2 Institute of Biomedical Engineering, College of Medicine, Southwest Jiaotong University, Chengdu 610031, China; ljsqaz0@163.com; 3 Key Laboratory of Advanced Technologies of Materials, Ministry of Education, School of Materials Science and Engineering, Southwest Jiaotong University, Chengdu 610031, China; laiyu572025615@163.com (Y.L.); liuzh993@126.com (Z.L.); 4 College of Materials Science and Engineering, Chongqing University of Technology, Chongqing 400054, China; wangxuanlun@cqut.edu.cn; 5 Research Institute of Frontier Science, Southwest Jiaotong University, Chengdu 610031, China

**Keywords:** energy harvesting, natural materials, self-powered nanosystems, transient electronics, wearable electronics

## Abstract

The rapid development of wearable electronics requires multifunctional, transient electronic devices to reduce the ecological footprint and ensure data security. Unfortunately, existing transient electronic materials need to be degraded in chemical solvents or body fluids. Here, we report green luminescent, water-soluble, and biocompatible piezoelectric nanofibers developed by electrospinning green carbon quantum dots (G-CQDs), mulberry silk fibroin (SF), and polyvinyl alcohol (PVA). The introduction of G-CQDs significantly enhances the piezoelectric output of silk fibroin-based fiber materials. Meanwhile, the silk fibroin-based hybrid fibers maintain the photoluminescent response of G-CQDs without sacrificing valuable biocompatibility. Notably, the piezoelectric output of a G-CQD/PVA/SF fiber-based nanogenerator is more than three times higher than that of a PVA/SF fiber-based nanogenerator. This is one of the highest levels of state-of-the-art piezoelectric devices based on biological organic materials. As a proof of concept, in the actual scenario of a rope skipping exercise, the G-CQD/PVA/SF fiber-based nanogenerator is further employed as a self-powered wearable sensor for real-time sensing of athletic motions. It demonstrates high portability, good flexibility, and stable piezoresponse for smart sports applications. This class of water-disposable, piezo/photoactive biological materials could be compelling building blocks for applications in a new generation of versatile, transient, wearable/implantable devices.

## 1. Introduction

Conventional electronics are usually extremely stable and can function properly even under extreme conditions [1,2,3,4]. This stability leads to difficult degradation of electronics after their obsolescence or destruction, making them one of the major pollutants in the environment [1,2,3,4]. The emerging transient electronics are electronic devices that can be partially or fully degraded in a short period of time after completing specified functions [1,2,3,4]. In particular, the degradation products of natural biopolymer-based transient electronics can be safely absorbed by the human body or the environment [4,5,6,7,8,9,10,11,12,13,14]. Therefore, they are attracting increasing attention in the fields of biomedicine, environmental protection, and information security [1,2,3,4,5,6,7,8,9,10,11]. Examples include bioabsorbable, implantable medical devices, environmentally friendly, degradable electronics, and self-destructive security hardware in military systems [1,2,3,4,5,6,7,8,9,10,11]. Unfortunately, most of the transient electronic materials currently available need to be degraded in chemical solvents or body fluids, which limits the range of applications of state-of-the-art transient electronic devices [1,2,3,4,5,6,7,8,9,10,11,12,13,14]. Moreover, it remains a great challenge for the design and manufacturing of versatile transient materials to meet the practical requirements of self-sustainable transient optoelectronics [3,4,6,7].

Mulberry silk is a natural animal protein fiber [14,15,16,17,18,19]. It is one of the earliest and most widely used biological polymer materials [14,15,16,17,18,19]. Mulberry silk is known as the “Queen of Fibers” because of its special luster, good air permeability, strong moisture absorption, and high mechanical strength [16,17,18,19]. The raw silk fiber in the silkworm cocoon consists of two parallel fibroin fibers with a thin layer of sericin on the surfaces [18,19,20]. Silk fibroin exhibits crystalline polymorphism, in which silk I has an alpha-helix structure with an orthorhombic unit cell, and silk II has an anti-parallel beta-sheet structure with a monoclinic unit cell [18,19,20,21]. Both crystal units lack a center of symmetry, and silk fibroin, like other bio-piezoelectric materials, shows intrinsic shear piezoelectricity [20,21]. Overall, a high degree of crystalline orientation is correlated with a strong piezoelectric effect in silk fibroin [20,21]. Natural silk fibroin-based materials are promising candidates for piezoelectric building blocks in physically transient, wearable/implantable biomedical devices because of their biocompatibility, biosafety, and environmental sustainability [14,15,16,17,18,19,20,21,22,23,24]. Nevertheless, compared with commonly used inorganic piezoelectric materials, the piezoelectric response is generally weak for silk fibroin-based materials [18,19,20,21]. Hence, recent advances in silk fibroin-based materials have focused on enhancing piezoelectric output without altering biocompatibility to develop practical transient devices [17,19,20].

Carbon-based quantum dots (CQDs) are a fascinating class of carbon nanomaterials with unique physiochemical, optoelectronic, and biological properties [10,25,26,27,28,29]. The emerging CQDs have demonstrated potential for a wide range of applications from electronic devices to photoelectrochemical devices, although the research is still in its early stages [10,25,26,27,28,29]. Here, we develop green luminescent, water-soluble, and biocompatible piezoelectric nanofibers by electrospinning green carbon quantum dots (G-CQDs), mulberry silk fibroin (SF), and polyvinyl alcohol (PVA), a biodegradable, hydrophilic polymer, as a co-spinning agent. The introduction of G-CQDs significantly enhances the piezoelectric output of silk fibroin-based fiber materials. The measured piezoelectric output of a G-CQD/PVA/SF fiber-based nanogenerator is more than three times higher than that of a PVA/SF fiber-based nanogenerator. This is one of the highest levels of state-of-the-art piezoelectric devices based on biological organic materials. As a proof of concept, in the actual scenario of a rope skipping exercise, the G-CQD/PVA/SF fiber-based transient device is further employed as a self-powered wearable sensor for real-time sensing of athletic motions. It demonstrates high portability, good flexibility, and stable piezoresponse for smart sports applications.

## 2. Experimental Section

### 2.1. Materials and Reagents

Mulberry silkworm cocoons were collected from silk-producing communities in Ankang, Shaanxi, China. Polyvinyl alcohol (PVA, grade 1788, alcoholysis degree 87.0–89.0%) was supplied by Macklin Biochemical Co., Ltd., Shanghai, China. Green emissive carbon quantum dots (G-CQDs) were provided by Janus New Materials Co., Ltd., Nanjing, China.

Lithium bromide (LiBr, analytical grade) was supplied by Macklin Biochemical Co., Ltd., Shanghai, China. Sodium carbonate (Na_2_CO_3_, analytical grade) was provided by D&B Biological Science and Technology Co., Ltd., Shanghai, China. Dialysis bags (MWCO 7000) were supplied by Hunan Yibo Biotechnology Co., Ltd., Changsha, China.

Mouse embryonic fibroblasts (NIH-3T3) were provided by China Cell Bank. Phosphate-buffered saline (PBS) was supplied by Titan Technology Co., Ltd., Shanghai, China. High glucose medium (DMEM) was provided by Thermo Fisher Scientific. Live and dead cell viability/toxicity detection kits were purchased from Jiangsu KeyGEN Biotech Co., Ltd., Nanjing, China. Cell counting kit-8 (CCK-8) was supplied by Beyotime Biotechnology Co., Ltd., Shanghai, China.

### 2.2. Electrospinning of G-CQD/PVA/SF Nanofibers

A schematic illustration of the fabrication process of electrospun G-CQD/PVA/CS nanofibers is shown in Figure S1a of the Supporting Information. The silkworm silk was boiled in a 0.02 M aqueous solution of Na_2_CO_3_, rinsed with water to remove the gum-like sericin protein, and dried overnight in an oven. The resultant degummed silk, i.e., silk fibroin (SF), was dissolved in a 9.3 M aqueous solution of LiBr at 60 °C for 4 h. The solution was dialyzed in deionized water for 48 h to extract LiBr. The silk fibroin solution was prepared with a concentration of 7.0 wt%.

Next, 2 g of PVA powder was dispersed in 5 mL of deionized water and stirred at 80 °C for 3 h. Then, 10 mL of SF solution and G-CQDs [0, 0.1, and 0.2%, w(CQD)/w(SF/PVA), respectively] were added to the above PVA solution. The mixture was stirred at 25 °C for 4 h and ultrasonicated for 30 min to obtain a homogeneous G-CQD/PVA/SF spinning solution. The G-CQD/PVA/SF-based electrospun nanofibers are denoted here as G-CQDs-x, and x indicates the mass fraction of G-CQDs with PVA/SF. For the nanofibers of G-CQDs-0, G-CQDs-1, and G-CQDs-2, the mass ratios of G-CQDs to PVA/SF are 0, 0.001, and 0.002, respectively.

Subsequently, the spinning solution was transferred to a 10 mL syringe with a 0.41 mm inner diameter of the spinneret. The solution was injected at a rate of 15 μL/min, and the applied voltage was 16 kV. The nanofibers were collected on an aluminum foil-covered, grounded, high-speed rotating drum. The distance between the spinning nozzle and the drum was 16 cm, and the drum’s rotating speed was 4000 rpm. Finally, the electrospun nanofiber films were removed from the drum and sealed in a dry environment.

### 2.3. Characterization of Materials

Scanning electron microscopy (SEM) was performed using a Zeiss Gemini 300 apparatus (Carl Zeiss AG, Oberkochen, Germany). Energy dispersive spectroscopy (EDS) was carried out with an Oxford Xplore 30 spectrometer (Oxford Instruments plc, Abingdon, UK). Transmission electron microscopy (TEM) images were acquired on a JEOL JEM-2100F instrument (JEOL Ltd., Tokyo, Japan). Laser scanning confocal microscopy (LSCM) was conducted using a Nikon A1R+ microscope (Nikon Corp., Tokyo, Japan). Attenuated total reflectance Fourier transform infrared (ATR-FTIR) spectra were recorded using a Nicolet iS50 spectrometer (Thermo Fisher Scientific, Inc., Waltham, MA, USA). X-ray diffraction (XRD) phase data were collected using a PANalytical X’pert PRO diffractometer (Malvern Panalytical Ltd., Malvern, UK). X-ray pole figures were measured using a PANalytical Empyrean Series 2 diffraction system (Malvern Panalytical Ltd., Worcestershire, UK) for texture analysis. Photoluminescence (PL) spectra were recorded using an Edinburgh FLS980 spectrometer (Edinburgh Instruments Ltd., Livingston, UK). The absolute PL quantum yields (QYs) were determined using a calibrated integrating sphere mounted on the spectrometer. The chromaticity coordinates were calculated using the 1931 CIE (International Commission on Illumination) system. The dielectric properties were tested using a Novocontrol Concept 41 broadband dielectric impedance spectrometer (Novocontrol Technologies GmbH & Co. KG, Montabaur, Germany).

### 2.4. Cytocompatibility of Hybrid Nanofibers

The hybrid fiber samples were prepared as squares with a side length of 0.5 cm and used for cellular experiments after being sterilized for 12 h by UV light. The cytotoxicity and biocompatibility of the hybrid fibers were assessed by CCK-8 assay and live/dead staining. The hybridized fibers were co-cultured with NIH-3T3 cells (2 × 10^4^ cells per well) at 37 °C in a 5% CO_2_ humidified incubator. The cell activity was detected using CCK-8 at 1 and 3 days of culture. The cells were stained with live/dead cell stain. The live/dead state of the cells was observed using a laser scanning confocal microscope.

### 2.5. Fabrication of PENGs

The G-CQDs-x-based piezoelectric nanogenerator (PENG) has a sandwich structure consisting of electrospun G-CQD/PVA/SF nanofiber film, a copper (Cu) electrode, and polyurethane (PU) tape. An electrospun nanofiber film with a thickness of 100 μm was cut into a square with a side length of 2.0 cm, and then two pieces of copper foil were attached to the upper and lower sides of the square fiber film. The copper foils were used as electrodes to derive the electric signals. The trilayer square was further encapsulated using transparent PU tape. The PU tape was used as a protective layer for the device. Finally, two acrylic plates were adhered to both sides of the PENG device. This ensured a continuous, smooth impact in the subsequent piezoelectric measurements.

### 2.6. Test of Devices

The piezoelectric performance of the G-CQDs-x-based PENGs was evaluated by measuring the electrical outputs under the stimulation of dynamic impulse forces. A schematic diagram of the impact measurement system is shown in Figure S1b of the Supporting Information. A linear motor of NTIAG HS01-37 × 166 was used as the impact source. The output voltage signals were measured using a Keithley 6514 system electrometer (Keithley Instruments, Inc., Cleveland, OH, USA). The output current signals were collected using a Stanford Research SR570 low-noise current amplifier (Coherent Scientific, Thebarton, Australia). The switching polarity test verified that the output signals truly came from the direct piezoelectric responses of G-CQDs-x-based devices (Figure S1c, Supporting Information) [30,31].

### 2.7. Monitoring of Human Physiology

The relevant biological signals were collected from the volunteers. Informed consent was obtained prior to the research. The relevant experiments involving human subjects conformed to the local ethical requirements.

## 3. Results and Discussion

**Figure 1:**
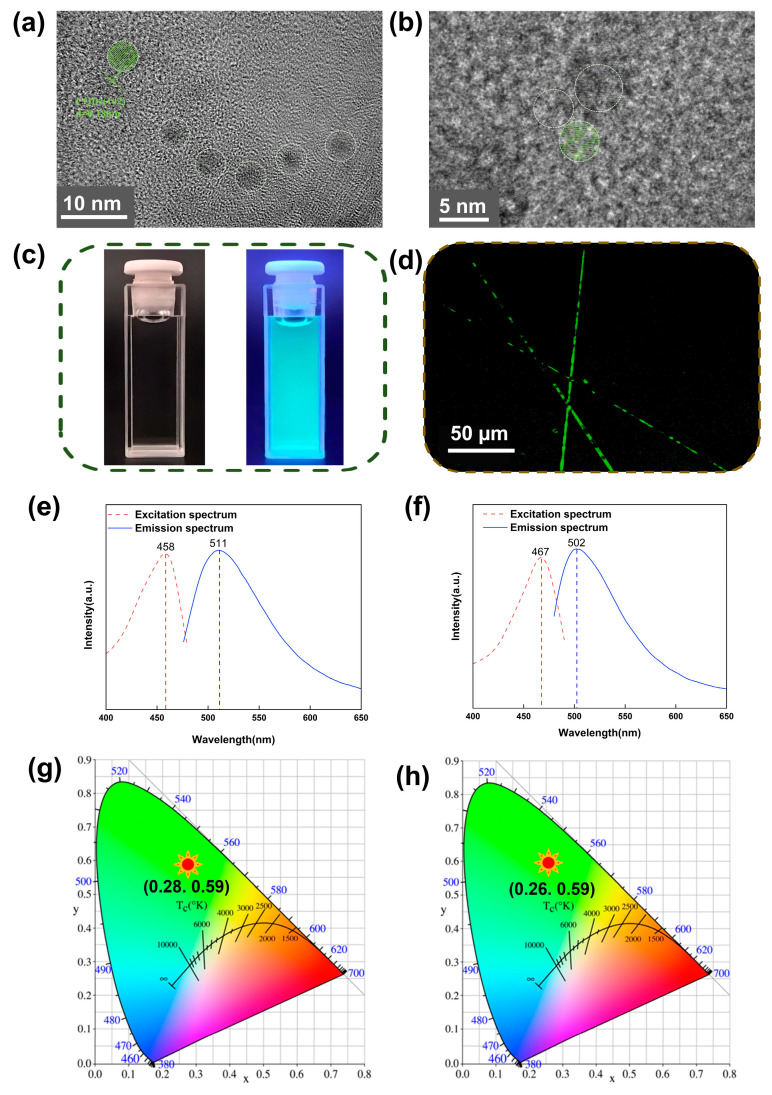
(**a**) TEM of G-CQDs in water. (**b**) TEM of G-CQDs in electrospun G-CQD/PVA/SF fibers. (**c**) Digital photographs of G-CQDs in water under natural light (left) and UV light (right). (**d**) Laser scanning confocal microscopy image of G-CQD/PVA/SF fibers. (**e**) PL excitation/emission spectra of G-CQDs. (**f**) PL excitation/emission spectra of G-CQD/PVA/SF. (**g**) CIE color coordinates of G-CQDs (λ_ex_: 458 nm). (**h**) CIE color coordinates of G-CQD/PVA/SF (λ_ex_: 467 nm).

The successful electrospinning of G-CQD hybridized silk fibroin-based nanofibers was confirmed using energy dispersive spectroscopy and transmission electron microscopy. The EDS mapping images clearly show the uniform distribution of elements in the electrospun G-CQD/PVA/SF nanofibers (Figure S2, Supporting Information). Besides C, O, and N elements, the electrospun nanofibers contain Zn, Si, S, Ca, and Sr elements. The abundant signals of element C mainly originate from the carbon skeleton of SF and PVA, and a small number of signals are associated with G-CQDs. The sources of element O include the molecular structures of PVA and SF, and the functional groups contained on the surfaces of G-CQDs, such as hydroxyl and carboxyl groups. The signals of element N indicate that the SF is rich in peptide bonds and nitrogen groups. The element S comes from sulfur-containing amino acids, e.g., cysteine and methionine, while the elements Zn, Si, Ca, and Sr represent the trace inorganic elements in SF [32,33]. Figure 1a,b shows the distribution and morphology of G-CQDs in water and in electrospun fibers, respectively. The individual G-CQDs are highly crystalline, spherical particles with a diameter of about 3 nm. They have clear lattice fringes with a spacing of 0.18 nm, corresponding to the d-spacing value of the graphene (102) plane in Powder Diffraction Files. The G-CQDs are monodispersed in water. They are readily able to self-organize in the matrix of hybrid fibers to form nanoscale assemblies.

The transparent G-CQD aqueous solution is colorless under natural light, and it emits green fluorescence under UV light irradiation (Figure 1c). The direct laser confocal microscopy observation definitely demonstrates that the G-CQD hybridized, silk fibroin-based electrospun nanofibers retain the photoluminescent response of G-CQDs (Figure 1d). Figure 1e,f shows the PL excitation/emission spectra of G-CQDs and G-CQD/PVA/SF, respectively. For G-CQDs, the optimal excitation wavelength is 458 nm, and the optimal emission wavelength is 511 nm (Figure 1e). For the G-CQD/PVA/SF mixture, the optimal excitation wavelength is red-shifted to 467 nm, and the optimal emission wavelength is blue-shifted to 502 nm, as compared to G-CQDs (Figure 1f). Based on the PL spectra, the corresponding CIE color coordinates of G-CQDs and G-CQD/PVA/SF are shown in Figure 1g,h, respectively. Obviously, the CIE color coordinates of green fluorescence remain almost the same, although the PL excitation and emission peaks of G-CQD/PVA/SF are red- and blue-shifted by 9 nm, respectively. This further confirms that the G-CQD/PVA/SF mixture maintains the photoluminescence of introduced G-CQDs. The absolute PL QY of the G-CQDs was determined to be 29.90% for green emission. Interestingly, the measured PLQY increased to 39.45% for the green emissive G-CQD/PVA/SF. Considering the red-shift excitation and blue-shift emission frequencies of G-CQD/PVA/SF, the enhancement of PL QY indicates the interactions between G-CQD surfaces, PVA molecules, and SF molecules [11,34,35,36].

**Figure 2:**
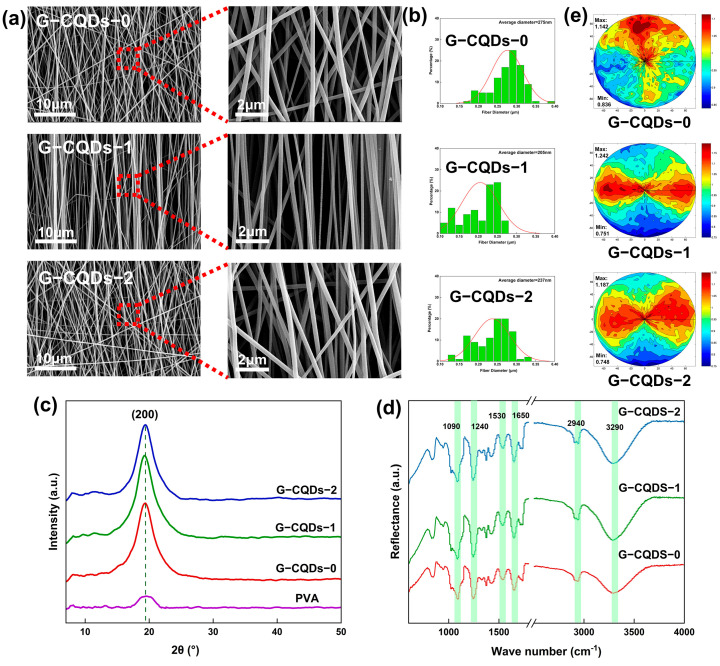
(**a**) SEM of G-CQDs-x fibers. (**b**) Diameter distribution of G-CQDs-x fibers. (**c**) XRD of G-CQDs-x fibers. (**d**) FTIR of G-CQDs-x fibers. (**e**) X-ray pole figures of the SF (200) plane in G-CQDs-x fibers.

The morphology, diameter, and alignment of electrospun nanofibers can be controlled by adjusting spinning parameters [37]. In general, the piezoelectric material-based electrospun fibers with smooth surfaces, small diameters, uniform morphology, and high orientation are beneficial for enhancing the piezoelectric response at a device level [9,10,11,37]. Figure 2a,b shows the SEM images and the diameter distribution of the G-CQD/PVA/SF-based electrospun nanofibers with various contents of G-CQDs. Evidently, the bead-less, smooth G-CQDs-x nanofibers were fabricated by optimizing spinning parameters (Figure 2a). Among them, the G-CQDs-1 fibers are highly preferentially oriented. Also, the G-CQDs-1 fibers have a smaller fiber diameter and a moderate distribution of diameters (Figure 2b). The morphological differences of G-CQDs-x fibers are mainly due to the inclusion of different amounts of G-CQDs. The inclusion of G-CQDs simultaneously increases the electrical conductivity and the viscosity of the spinning solution [9,10,37]. During electrospinning, the coupling and competition between conductivity and viscosity determine the final fiber morphology of electrospun nanofibers [9,10,37].

Figure 2c shows the XRD diffractograms of pristine PVA-based nanofibers and different G-CQD/PVA/SF-based nanofibers. The intense diffraction peak at a 2θ angle of 19.2°, corresponding to the (200) lattice plane of silk II, β-sheet crystals [33,38,39], definitely confirms the highly crystalline nature of SF in the G-CQDs-x-based electrospun nanofibers. Figure 2d shows the IR spectra of different G-CQDs-x fibers. The three characteristic peaks at 1650, 1530, and 1240 cm^−1^ correspond to amide I (C=O stretching), amide II (N-H bending), and amide III (C-N stretching) bands of silk fibroin, respectively [33,40]. This indicates the retention of the secondary structure of silk fibroin in the electrospun hybrid fibers [33,40]. The characteristic absorption peaks at 3290, 2940, and 1090 cm^−1^ are attributed to O-H, C-H, and C-OH stretching vibrations of polyvinyl alcohol, respectively [41]. Thus, the initial characteristic peaks of SF and PVA were all detected in the hybridized fibers. Moreover, for different G-CQDs-x fibers, no obvious changes were observed in these characteristic peaks, even with the increase of G-CQD contents. This suggests that there is no strong covalent interaction between G-CQDs, PVA, and SF in the hybrid systems. They might interact with each other through intermolecular interactions such as hydrogen bonding and van der Waals forces [33,40,41].

Figure 2e shows the measured X-ray pole figures of the SF (200) plane in different G-CQDs-x fibers. For crystalline materials, a pole figure is a direct visualization of crystal orientation at a given Bragg angle [42,43]. Clearly, each pole figure reveals the presence of polar angle-dependent pole density. This confirms a preferential crystal orientation rather than a random crystal distribution in each G-CQDs-x sample. In particular, the highly aligned G-CQDs-1 nanofiber sample exhibits the highest maximum pole density. This actually indicates the highest level of preferential crystalline orientation of internal piezo-phase SF lamellae [42,43]. Therefore, the inclusion of an appropriate amount of G-CQDs enables the electrospinning of a double-aligned, green luminescent silk fibroin-based fibrous material.

**Figure 3:**
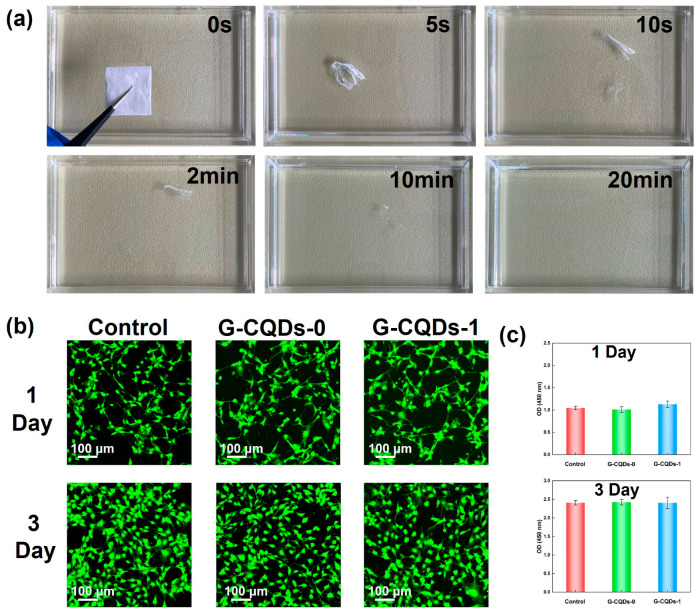
(**a**) Digital pictures showing that G-CQDs-1 fiber film fully dissolves after submerging in water for 20 min. (**b**) Fluorescence images of NIH-3T3 cells on the control, G-CQDs-0, and G-CQDs-1 for 1 day. Fluorescence images of NIH-3T3 cells on the control, G-CQDs-0, and G-CQDs-1 for 3 days. (**c**) Cell proliferation on the control, G-CQDs-0, and G-CQDs-1 for 1 day and 3 days of culture.

Figure 3a shows digital pictures of a G-CQDs-1 fiber-based film at various stages of dissolution in water. Notably, the free-standing fiber film was fully dissolved in water, and it disappeared within 20 min at room temperature. Room-temperature water-disposable piezoelectric fibers are particularly attractive for waste-free wearable/implantable electronics to ensure the security of personal data. Hence, cytotoxicity and biocompatibility, two important parameters in assessing the biosafety of biorelevant materials [44], were further investigated for these G-CQD hybridized, silk fibroin-based fibrous materials. NIH-3T3 cells are a mouse embryonic fibroblast cell line that is widely used in biomedical research. Due to their stable proliferation, easy culture conditions, and reproducible experimental results, NIH-3T3 cells have been widely used for in vitro cellular experiments [45,46]. As shown in Figure 3b, most of the NIH-3T3 cells were stained green. The NIH-3T3 cells grown for 1 day showed isolated, round shapes. In contrast, the NIH-3T3 cells grown for 3 days were more densely packed. They had a more pike-shaped morphology and were in good cellular condition. In addition, according to the results of CCK-8 analysis in Figure 3c, the cell proliferation ability of the treated G-CQDs-x group was basically the same as that of the control group. Consequently, the silk fibroin/carbon quantum dot-based nanofiber materials demonstrate good biocompatibility.

**Figure 4:**
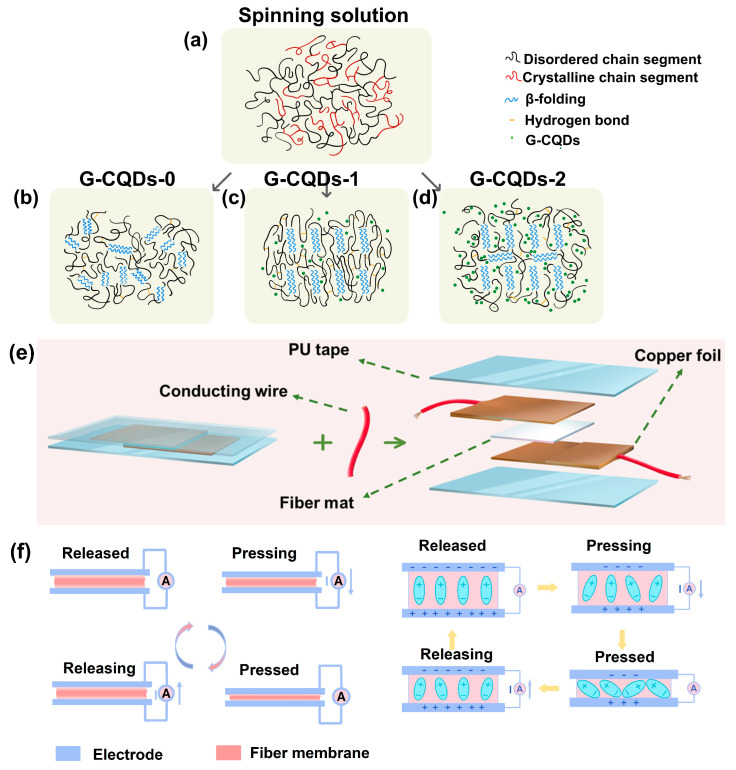
(**a**–**d**) Schematic diagrams illustrating the microstructure of SF in (**a**) spinning solution, and in electrospun (**b**) G-CQDs-0, (**c**) G-CQDs-1, and (**d**) G-CQDs-2 fibers. (**e**) Schematic drawing of the composite structure of G-CQDs-x fiber-based piezoelectric device. (**f**) Schematic illustrations of the working mechanism of the G-CQDs-x fiber-based piezoelectric device in a macroscopic state (**left**) and in a microscopic state (**right**).

The measured X-ray pole figures indicate the different orientations of piezoelectric phase SF crystals in different G-CQDs-x fiber samples. Figure 4a–d shows a schematic illustration of the orientation growth of piezo-phase silk fibroin lamellae in different electrospun G-CQDs-x fibers. Initially, the disordered molecular chains of silk fibroin are randomly distributed in the spinning solution (Figure 4a). During electrospinning, silk fibroin begins to crystallize under the action of electrostatic forces [11,37,47]. The electric field-induced elongation strain enables the partially oriented growth of silk fibroin lamellae, even if the G-CQDs are not included (Figure 4b). Notably, the inclusion of a very small yet moderate amount of G-CQDs allows the highly preferentially oriented growth of silk fibroin lamellae (Figure 4c). However, excessive inclusion of G-CQDs results in the misoriented growth of silk fibroin lamellae (Figure 4d).

The dielectric constant of different electrospun G-CQDs-x fibers was measured over a wide range of frequencies (Figure S3, Supporting Information). The dielectric constant of G-CQDs-x fibers decreases with increasing frequency, which is a well-known property of piezoelectric materials and well supports the piezoelectric nature of these G-CQDs-x fibers. Moreover, the change of dielectric constant is consistent with the change of piezo-phase SF crystalline orientation in these G-CQDs-x fibers. Moderate addition of G-CQDs promotes the preferential orientation growth of piezo-phase SF lamellae, enhances the polarization response, and ultimately improves the dielectric constant. This makes the G-CQD hybridized silk fibroin an excellent piezoelectric material for developing physically transient, self-powered nanosystems. Hence, the mechanically flexible, free-standing electrospun G-CQDs-x fiber films were further assembled into prototype PENG devices to evaluate the device-level piezoelectric properties (Figure 4e).

Figure 4f illustrates schematically the operation mechanism of the green-emission, water-soluble biofiber-based piezoelectric device in a macroscopic state and a microscopic state. The structural origins of piezoelectricity in the silk fibroin-based fiber films are the molecular dipoles in the uniaxially aligned, non-centrosymmetric SF crystalline lamellae [20,21,48,49,50,51]. Initially, the G-CQDs-x-based device remains in a state of polarization equilibrium. Under the stress-free condition, no piezoelectric signal is produced. When the device is pressed, the aligned dipoles move with the deformation of aligned SF crystals. The polarization level decreases, and the free charges flow between electrode surfaces. This results in an instantaneous piezoelectric signal. The release of the applied stress allows for the recovery of SF crystals to their original positions. The molecular dipoles move backward, and the polarization level increases. As a consequence, the charges flow back to balance the surface charge. This induces a reverse piezoelectric signal. In this way, the stable, durable operation of the PENG device is realized using the direct piezoresponse of G-CQDs-x-based biofibers.

**Figure 5:**
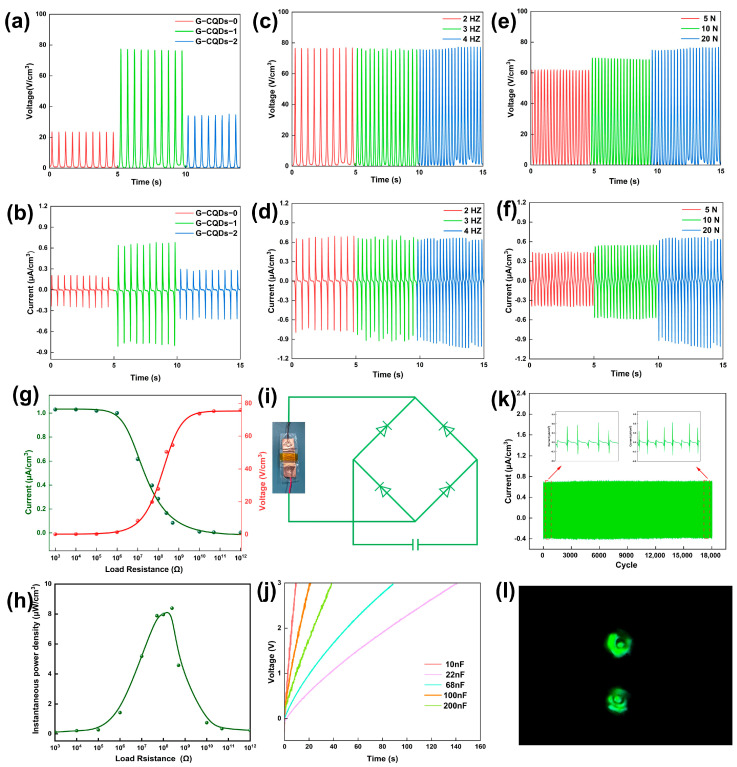
(**a**) Open-circuit voltage and (**b**) short-circuit current of G-CQDs-x devices, with different G-CQD contents, stimulated using a 20 N, 2 Hz impulse force. (**c**) Open-circuit voltage and (**d**) short-circuit current of the G-CQDs-1 device, stimulated using a fixed impulse force of 20 N and at different frequencies. (**e**) Open-circuit voltage and (**f**) short-circuit current of the G-CQDs-1 device, stimulated at varying impulse forces and at a fixed frequency of 4.0 Hz. (**g**) Output voltage and output current, and (**h**) peak power density of the G-CQDs-1 device as a function of external load resistance, measured under a 20 N, 4 Hz impulse force. (**i**) Equivalent circuit for charging a commercial capacitor. The inset shows a digital picture of the G-CQDs-1 device. (**j**) Charging curves of different capacitors using the G-CQDs-1 device. (**k**) Cyclic stability test of the G-CQDs-1 device for 18,000 cycles, whole signal, under the stimulation of a 10-N, 3-Hz impulse force. (**l**) Digital image showing the instant glow of green LEDs using the power generated by the G-CQDs-1 device under finger tapping.

Figure 5a,b shows the piezoelectric output of the G-CQDs-x-based nanogenerators, with different mass ratios of G-CQDs to PVA/SF, stimulated using a 20 N, 2 Hz impulse force. As expected, the piezoelectric output is proportional to the orientation level of the piezoelectric phase SF lamellae in the G-CQDs-x-based fibers. The output voltage and output current of the G-CQDs-1-based device reach 72 V cm^−3^ and 1.01 μA cm^−3^, respectively. The piezoelectric output is more than three times higher than that of a G-CQDs-0-based device. The piezoelectric characteristics of the G-CQDs-1-based device were further evaluated by varying the amplitude of the impulse force and stimulating frequency (Figure 5c–f). The device shows stress- and frequency-dependent piezoelectric output. Nevertheless, the piezoelectric output is more sensitive to the change in the amplitude of the impulse force. Under a 20 N, 4 Hz impulse force, the output voltage and output current reach 76 V cm^−3^ and 1.03 μA cm^−3^, respectively. The measured piezoelectric output is one of the highest levels of state-of-the-art piezoelectric devices based on biological organic materials (Table S1, Supporting Information) [52,53,54,55,56,57,58,59,60].

Figure 5g,h shows the output voltage, output current, and peak power density of a G-CQDs-1-based nanogenerator under different external load resistances. The piezoelectric device was stimulated using a 20 N, 4 Hz impulse force. The measured piezoelectric power density reaches a maximum value of 8.39 μW cm^−3^ at an external load resistance of 250 MΩ. Using the generated electricity, the G-CQDs-1-based device is capable of charging different commercial capacitors within a relatively short time (Figure 5i,j). Moreover, in over 18,000 continuous operation cycles, the bio-based device demonstrates extraordinary piezoelectric stability, piezoelectric durability, and piezoelectric repeatability (Figure 5k). Under finger tapping, it is able to directly operate commercial light-emitting diode (LED) bulbs (Figure 5l). Hence, the measured piezoelectric characteristics definitely confirm the feasibility of the G-CQD hybridized, SF-based piezoelectric devices in practical applications.

**Figure 6:**
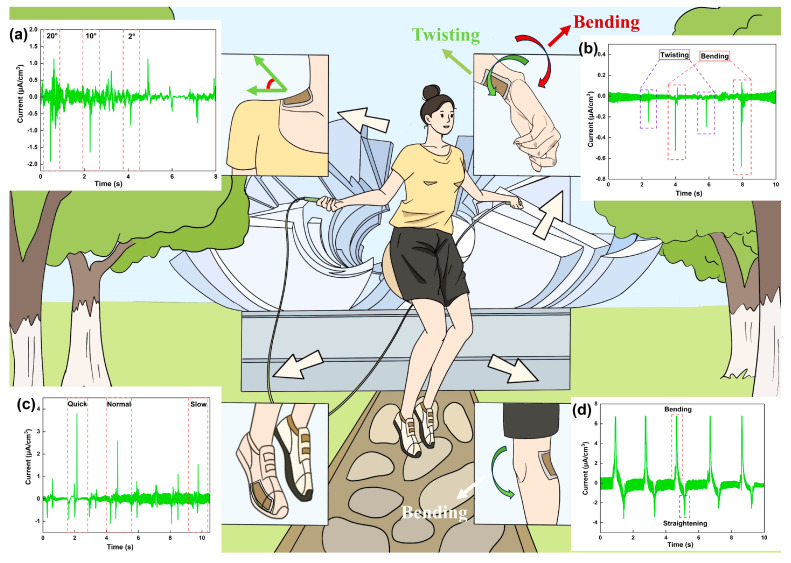
Demonstration of the G-CQDs-1 fiber-based piezoelectric device as a self-powered flexible wearable sensor for smart sports applications in the actual scenario of a rope skipping exercise. By attaching to different parts of the human body, it is able to precisely sense various human movement signals in real time: (**a**) human neck motion, (**b**) human wrist bending/twisting, (**c**) human knee-joint movement, (**d**) human foot pressing. Device size: 20 mm × 20 mm × 0.1 mm.

As a proof of concept, in the actual scenario of a rope skipping exercise, the mechanically flexible, shape-adaptable G-CQDs-1-based PENG device was employed as a self-powered tactile sensor for the real-time monitoring of athletic motions (Figure 6). In the rope skipping exercise, with the upper arm close to the body, the athlete keeps the shoulder relaxed and uses the forearm and wrist to rotate the rope. Then, the athlete bends the knee, lifts the front foot off the ground, and completes a rope skipping movement. By attaching the device to desirable parts of the human body, it is able to precisely recognize the different characteristic athletic motion signals during the rope skipping exercise. Examples include the key piezo signals produced by human neck motion (Figure 6a), human wrist bending/twisting (Figure 6b), human knee-joint movement (Figure 6c), and human foot pressing (Figure 6d). Hence, the water-soluble biofiber-based piezoelectric device meets the practical requirements of high sensitivity, high flexibility, and high reliability in sensing a broad range of biophysiological stresses and strains. It shows great potential for application in emerging intelligent wearable electronics [61,62].

## 4. Conclusions

In summary, we have successfully developed a novel class of versatile, transient, and bio-piezoelectric materials based on electrospun G-CQD/PVA/SF nanofibers. The striking merits included green luminescence, water solubility, good biocompatibility, and enhanced bio-piezoelectricity. The introduction of G-CQDs remarkably improved the piezoelectric output of silk fibroin-based fiber materials. Meanwhile, the silk fibroin-based hybrid fiber materials retained the PL response of G-CQDs without sacrificing valuable biocompatibility. Notably, under a 20 N, 2 Hz impulse force, the piezoelectric output of a G-CQD/PVA/SF fiber-based device was more than three times higher than that of a PVA/SF fiber-based device. The measured piezoelectric output is one of the highest levels of the state-of-the-art piezoelectric devices based on biological organic materials. The green-emission, water-soluble biofiber-based piezo device could be used as a piezoelectric nanogenerator for fast charging of commercial capacitors and direct powering of LED bulbs. Moreover, the transient piezo device could serve as a self-powered wearable sensor for the real-time sensing of athletic motions. It demonstrated high portability, good flexibility, and stable piezoresponse for smart sports applications. We believe this study may be beneficial for developing a new generation of versatile transient materials/devices for applications in motion monitoring, smart wearables, medical healthcare, and information security.

## Data Availability

Data will be made available on request.

## Supplementary Materials

The following supporting information can be downloaded at: https://www.mdpi.com/article/10.3390/polym17111579/s1, Figure S1: (a) Schematic diagram of the fabrication process of electrospun G-CQDs/PVA/SF nanofibers. (b) Schematic illustration of an impact measurement system for collecting piezoelectric outputs. (c) Voltage signals and (d) current signals of a transient bio-piezoelectric nanogenerator based on electrospun G-CQDs/PVA/SF nanofibers. The device is connected to the measurement system in forward (left) and reverse (right) connections, respectively; Figure S2: Typical EDS mapping images of electrospun G-CQDs/PVA/SF nanofibers showing elemental distribution of C, O, N, Zn, Si, S, Ca and Sr, respectively; Figure S3: Dielectric constant of electrospun G-CQDs-x fibers; Table S1: A comparison of the piezoelectric output of G-CQDs/PVA/SF based device with the recently reported bioorganic materials based piezoelectric nanogenerators.
