# Supplementary material for: Toward Versatile Transient Electronics: Electrospun Biocompatible Silk Fibroin/Carbon Quantum Dot-Based Green-Emission, Water-Soluble Piezoelectric Nanofibers
Source: Polymers (Basel). 2025 Jun 5;17(11):1579. doi: 10.3390/polym17111579 (PMC12157144; doi:10.3390/polym17111579)
Supplement: Supplementary file 1 [file polymers-17-01579-s001.zip › polymers-3638394-supplementary.pdf]

Supporting Information

Toward Versatile Transient Electronics: Electrospun Biocompatible Silk Fibroin/ Carbon Quantum Dot-Based Green-Emission, Water-Soluble Piezoelectric Nanofibers

Zhipei Xia [1], Chubao Liu [2, 3], Juan Li [1], Biyao Huang [1], Chu Pan [1], Yu Lai [3], Zhu Liu [3], Dongling Wu [1], Sen Liang [1], Xuanlun Wang [4], Weiqing Yang [5, *] and Jun Lu [1, *, †]

[1] School of Chemistry, Southwest Jiaotong University, Chengdu 610031, China; 13981794626@163.com (Z.X.); 18202840217@163.com (J.L.); 13164627201@163.com (B.H.); panchu@my.swjtu.edu.cn (C.P.); wdl4186@my.swjtu.edu.cn (D.W.); 15034167632@163.com (S.L.)

[2] Institute of Biomedical Engineering, College of Medicine, Southwest Jiaotong University, Chengdu 610031, China;
ljsqaz0@163.com

[3] Key Laboratory of Advanced Technologies of Materials, Ministry of Education, School of Materials Science and Engineering, Southwest Jiaotong University, Chengdu 610031, China;
laiyu572025615@163.com (Y.L.); liuzh993@126.com (Z.L.)

[4] College of Materials Science and Engineering, Chongqing University of Technology, Chongqing 400054, China;
wangxuanlun@cqut.edu.cn

[5] Research Institute of Frontier Science, Southwest Jiaotong University, Chengdu 610031, China

[*] Correspondence: wqyang@swjtu.edu.cn (W.Y.); junlyuprc@hotmail.com

[†] (J.L.) Other used names Jun Lv and Jun Lyu.

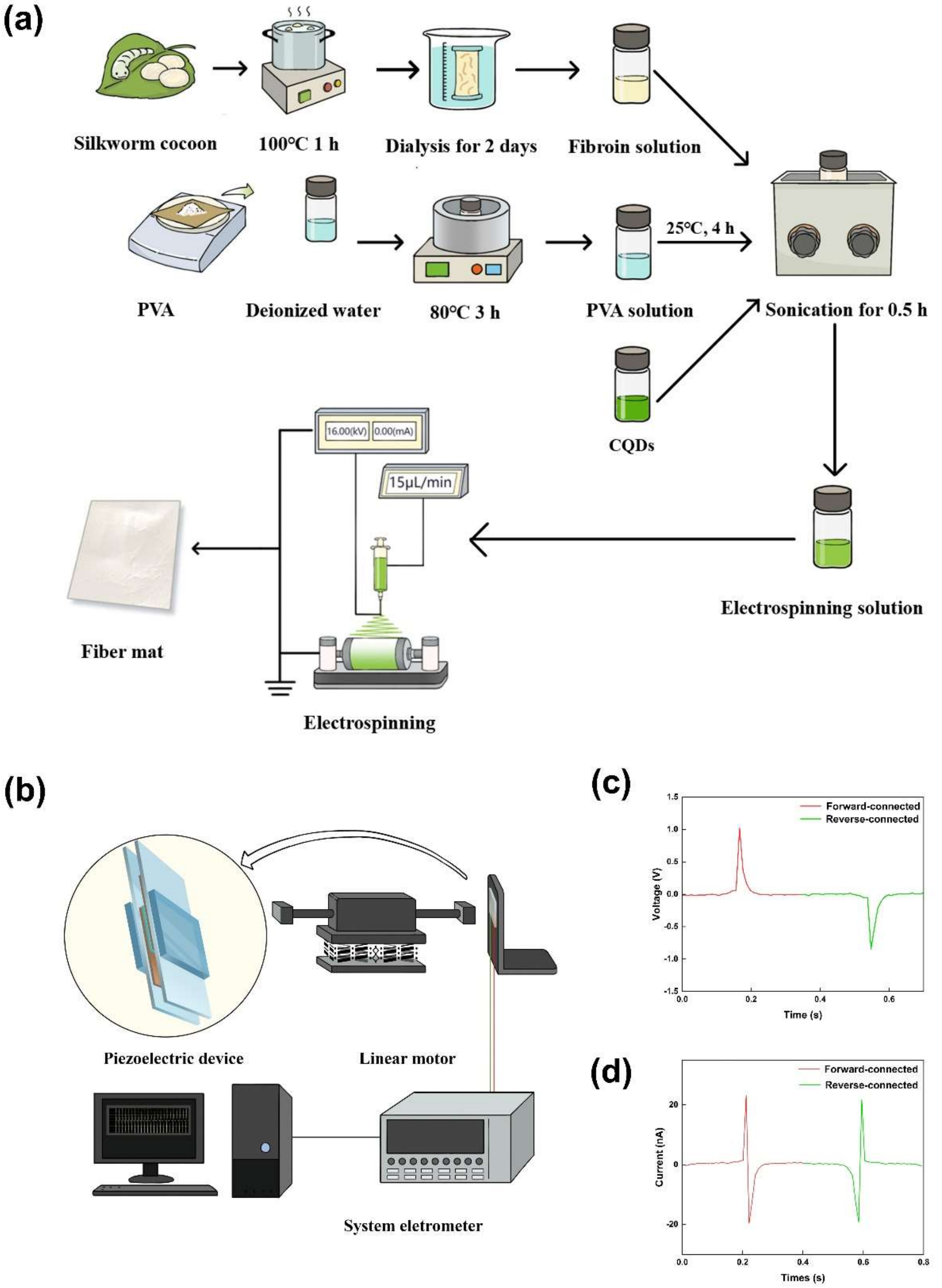

Figure S1. (a) Schematic diagram of the fabrication process of electrospun G-CQDs/PVA/SF nanofibers. (b) Schematic illustration of an impact measurement system for collecting piezoelectric outputs. (c) Voltage signals and (d) current signals of a transient bio-piezoelectric nanogenerator based on electrospun G-CQDs/PVA/SF nanofibers. The device is connected to the measurement system in forward (left) and reverse (right) connections, respectively.

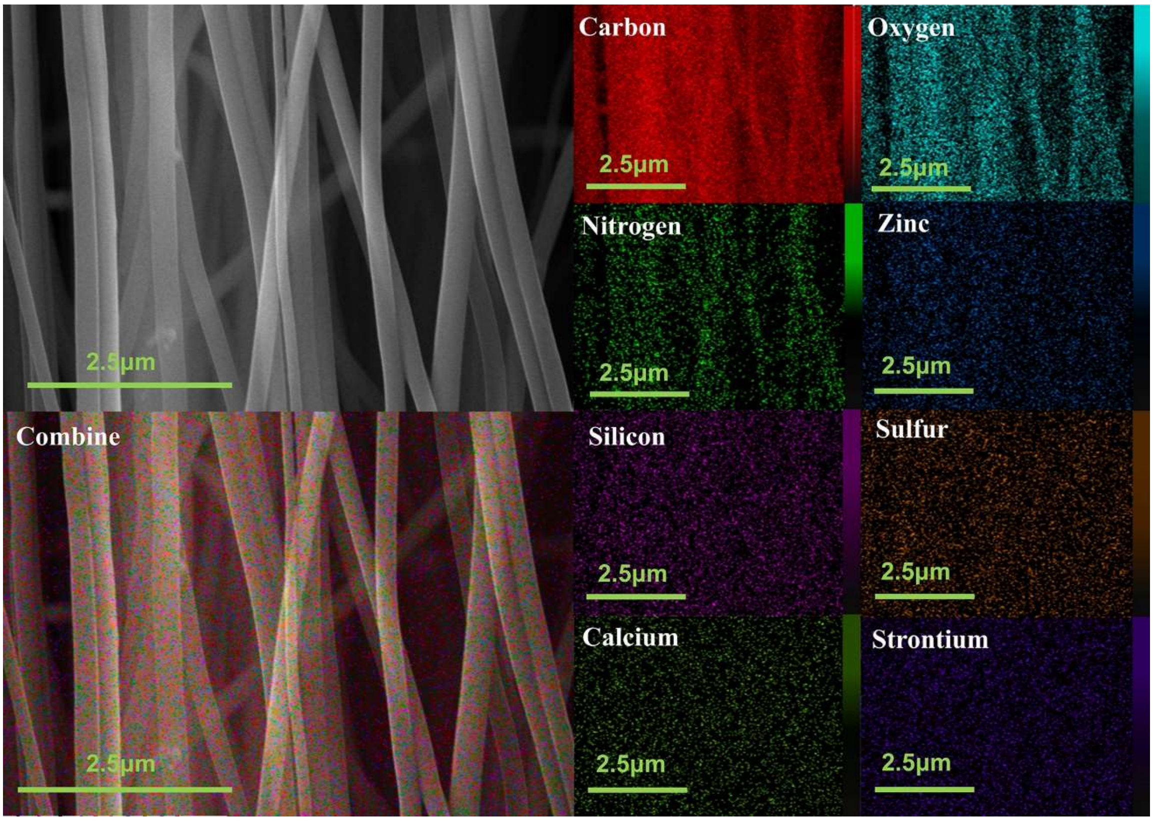

Figure S2. Typical EDS mapping images of electrospun G-CQDs/PVA/SF nanofibers showing elemental distribution of C, O, N, Zn, Si, S, Ca and Sr, respectively.

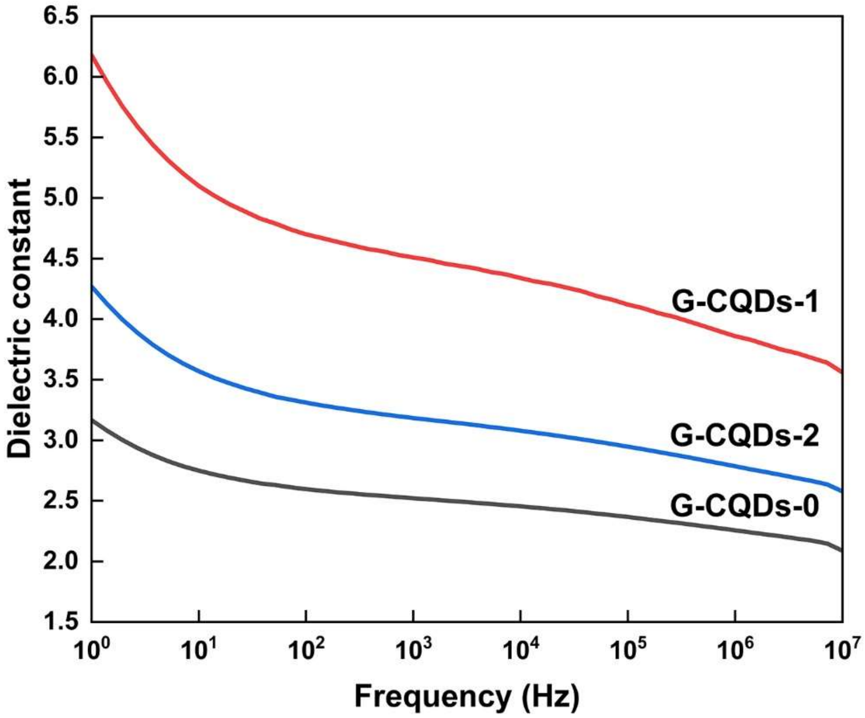

Figure S3. Dielectric constant of electrospun G-CQDs-x fibers.

Table S1. A comparison of the piezoelectric output of G-CQDs/PVA/SF based device with the recently reported bioorganic materials based piezoelectric nanogenerators.

No.	Material	Electrode	Areal size (mm^2)	Thickness (μm)	Voltage (V)	Current (nA)	Voltage density [a] ($V\ cm^{-3}$)	Current density [a] ($\mu A\ cm^{-3}$)	Ref.
1	Onion skin	Au	30 × 20	240	18 V	166	125	1.15	[1]
2	Aloe vera	Au/Al	40 × 40	27	1.19	547	27.55	12.66	[2]
3	Diphenylalanine nanotubes	Au	NF	0.415	2.8 V	37.4	—	—	[3]
4	Roasted wood	Cu	15 × 15	15000	1.4	14.5	0.41	0.0043	[4]
5	CNC	CNC-CNT	20 × 20	1200	15	200	31.25	0.42	[5]
6	Peptide	Au	12 × 12	75	0.6	7	55.56	0.65	[6]
7	CRC/ZnO	Au	22 × 32	NF	10.02	1032.57	—	—	[7]
8	ZnO/cellulose	Au	NF	40	1.5	80	—	—	[8]
9	CNF/ MXene/ ZnO	Al	25 × 18	210	17.15	997	181.48	10.55	[9]
**10**	**G-CQDs/PVA/SF**	**Cu**	**20 × 20**	**100**	**3.04**	**41.21**	**76**	**1.03**	**Present work**

[a] Values calculated based on other parameters reported in the references.

References

[1] S. Maiti, S. K. Karan, J. Lee, A. K. Mishra, B. B. Khatua and J. K. Kim, Bio-waste onion skin as an innovative nature-driven piezoelectric material with high energy conversion efficiency, *Nano Energy*, 2017, **42**, 282-293.

[2] N. R. Alluri, N. P. M. J. Raj, G. Khandelwal, V. Vivekananthan and S.-J. Kim, Aloe vera: A tropical desert plant to harness the mechanical energy by triboelectric and piezoelectric approaches, *Nano Energy*, 2020, **73**, 104767.

[3] J. H. Lee, K. Heo, K. Schulz-Schnhagen, J. H. Lee, M. S. Desai, H. E. Jin and S. W. Lee, Diphenylalanine peptide nanotube energy harvesters, *ACS Nano*, 2018, **12**, 8138-8144.

[4] T. Wu, Y. Lu, X. Tao, P. Chen, Y. Zhang, B. Ren, F. Xie, X. Yu, X. Zhou and D. Yang, Superelastic wood-based nanogenerators magnifying the piezoelectric effect for sustainable energy conversion, *Carbon Energy*, 2024, **6**, e561.

[5] K. Maity, A. Mondal and M. C. Saha, Cellulose nanocrystal-based all-3D-printed pyro-piezoelectric nanogenerator for hybrid energy harvesting and self-powered cardiorespiratory monitoring toward the human–machine interface, *ACS Appl. Mater. Interfaces*, 2023, **15**, 13956–13970.

[6] K. Jenkins, S. Kelly, V. Nguyen, Y. Wu and R. Yang, Piezoelectric diphenylalanine peptide for greatly Improved flexible nanogenerators, *Nano Energy*, 2018, **51**, 317-323.

[7] X. Song, H. Zou, S. Cao, B. Jiang, M. Li, L. Huang, Y. Zhang and Q. Yuan, Flexible regenerated cellulose/ZnO based piezoelectric composites fabricated via an efficient one-pot method to load high-volume ZnO with assistance of crosslinking, *Chem. Eng. J.*, 2023, **475**, 146184.

[8] G. Zhang, Q. Liao, M. Ma, F. Gao, Z. Zhang, Z. Kang and Y. Zhang, Uniformly assembled vanadium doped ZnO microflowers/bacterial cellulose hybrid paper for flexible piezoelectric nanogenerators and self-powered sensors, *Nano Energy*, 2018, **52**, 501-509.

[9] Q. Zhu, X. Chen, D. Li, L. Xiao, J. Chen, L. Zhou, J. Chen and Q. Yuan, Large enhancement on performance of flexible cellulose-based piezoelectric composite film by welding CNF and MXene via growing ZnO to construct a "brick-rebar-mortar" structure, *Adv. Funct. Mater.*, 2024, **34**, 2408588.
